# “*I had the feeling that I was trapped*”: a bedside qualitative study of cognitive and affective attitudes toward noninvasive ventilation in patients with acute respiratory failure

**DOI:** 10.1186/s13613-019-0608-6

**Published:** 2019-12-02

**Authors:** Marina Iosifyan, Matthieu Schmidt, Amélie Hurbault, Julien Mayaux, Christian Delafosse, Marina Mishenko, Nathalie Nion, Alexandre Demoule, Thomas Similowski

**Affiliations:** 1 UMRS1158 Neurophysiologie Respiratoire Expérimentale et Clinique, INSERM, Sorbonne Université, 75005 Paris, France; 2 Service de Pneumologie, Médecine Intensive et Réanimation, Département R3S, AP-HP, Groupe Hospitalier Pitié-Salpêtrière Charles Foix, 47-83 boulevard de l’Hôpital, 75013 Paris, France; 3 Department of Psychology, National Research University Higher School of Economics, Moscow, Russia; 4 Service de Réanimation Médicale de l’Institut de Cardiologie, AP-HP, Groupe Hospitalier Pitié-Salpêtrière Charles Foix, 75013 Paris, France; 5 Service de Pneumologie-Explorations du Sommeil, Hôpital Simone Veil, 95600 Eaubonne, France; 6 Laboratoire Psychopathologie et Processus de Santé, EA 4057, Université Paris Descartes, 75005 Paris, France; 7 Laboratoire de psychologie du développement et de l’éducation de l’enfant, UMR 8240, CNRS, Université Paris Descartes & Université Caen Basse-Normandie, 75005 Paris, France

**Keywords:** Noninvasive ventilation (NIV), Qualitative research, Cognitive attitudes, Affective attitudes, Dyspnea

## Abstract

**Background:**

Noninvasive ventilation (NIV) is the application of mechanical ventilation through a mask. It is used to treat certain forms of acute respiratory failure in intensive care units (ICU). NIV has clinical benefits but can be anxiogenic for the patients. This study aimed at describing cognitive and affective attitudes toward NIV among patients experiencing NIV for the first time in the context of an ICU stay.

**Methods:**

Semi-structured interviews were conducted in 10 patients during their ICU stay and soon after their first NIV experience. None of the patients had ever received NIV previously. Evaluative assertion analysis and thematic analysis were used to investigate cognitive and affective attitudes toward NIV before, during, and after the first NIV experience, as well as patient attitudes toward caregivers and relatives.

**Results:**

Before their first NIV session, the cognitive attitudes of the patients were generally positive. They became less so and more ambiguous during and after NIV, as the patients discovered the actual barriers associated with NIV. Affective attitudes during NIV were more negative than affective attitudes before and after NIV, with reports of dyspnea, anxiety, fear, claustrophobic feelings, and reactivation of past traumatic experiences. The patients had more positive attitudes toward the presence of a caregiver during NIV, compared to the presence of a family member.

**Conclusion:**

This study corroborates the possibly negative—or even traumatic—nature of the NIV experience, with emphasis on the role of affective attitudes. This is a rationale for evaluating the impact of NIV-targeted psychological interventions in ICU patients with acute respiratory failure.

## Background

Mechanical ventilation (MV) is a lifesaving therapeutic procedure routinely administered in intensive care units (ICUs). In certain circumstances [1], MV can be administered through a face mask, a procedure termed noninvasive ventilation (NIV) because it does not involve the insertion of an endotracheal device. Appropriately used, NIV brings important clinical benefits [2–4]. NIV also has its specific complications (e.g., facial skin lesions due to the pressure exerted by the mask) and can be perceived as a stressful experience: up to one-third of patients treated by NIV for acute respiratory failure associate it with high levels of anxiety [5]. Studies conducted in other contexts show that certain barriers to NIV can compromise adherence to treatment and even lead to its refusal [6]. Such barriers include fear of the mask, anxiety, claustrophobia, and dyspnea [6, 7]. Dyspnea under NIV is a complex issue. Indeed, NIV aims at correcting gas exchange, but it is also known to alleviate dyspnea and is presented as such to the patients. Should NIV fail to relieve dyspnea—or, worse, should dyspnea worsen during NIV—the conjunction of a vital threat with a feeling of lack of control is bound to aggravate both dyspnea and anxiety [8–10], thereby creating a traumatic vicious circle. Such suffering is heightened if it is not met by adequate attention from caregivers—invisible dyspnea or occult respiratory suffering—[11–14]. A focus on patients’ NIV-related experiences is therefore highly pertinent to successful use of the technique.

Attitudes toward NIV for acute respiratory failure differ between ICU physicians, ICU nurses, the patients, and their relatives [5]. The “PARVENIR” study, a prospective multicenter study conducted in French and Belgian ICUs [5], showed that physicians tend to consider NIV more positively than nurses, even though both professions agree that the treatment is stressful for the patients [5]. Patient attitudes are mixed: they reported considering NIV “pleasant” more often than nurses and “traumatic” less often than nurses, but at the time of ICU discharge, about one-third expressed regret that they had received NIV rather than having been intubated [5]. This is why we conducted the present research, as an addition to the PARVENIR study, with the aim of better understanding the determinants of the patients’ final regretful opinion and to refine our knowledge of patients’ cognitive representations of NIV (utility and efficacy) and their affective associations (feelings toward NIV). We were specifically interested in how cognitive and affective attitudes toward NIV evolve before, during, and after its administration. We also aimed at describing the attitudes of patients to the role of caregivers and relatives during NIV. This was done according to the principles of qualitative research, namely a scientific method of observation aiming at gathering and interpreting non-numerical data pertaining to how a given phenomenon is lived and perceived, rather than to a quantitative measure of this phenomenon. The present report therefore conforms with the COREQ guidelines for reporting qualitative studies [15]. Quantitative language analysis was also performed.

## Methods

### Context and ethical approval

The study was approved as ancillary to the PARVENIR study [5] by the Co*mité de Protection des Personnes Ile*-*de*-*France 6* (ethical committee). The patients gave written, informed consent to participate.

### Patients

Participants were recruited within a 16-bed ICU at a tertiary 1600-bed university hospital (Pitié-Salpêtrière Hospital, Paris, France). Inclusion criteria were: (1) age over 18; (2) first ICU admission for acute respiratory failure with a decision to initiate NIV by the physician in charge; (3) second or third day of NIV during the ICU stay; (4) no history of previous intubation; (5) no history of NIV before this ICU stay (including home mechanical ventilation). Exclusion criteria were: (1) delirium or confusion according to the Confusion Assessment Method for the Intensive Care Unit (CAM-ICU) [16]; (2) refusal to participate or incapacity to sign informed consent; (3) speech impediment (e.g., bulbar syndrome in patients with amyotrophic lateral sclerosis); (4) insufficient command of French.

We planned to recruit 10 patients. Thirteen consecutive patients were screened but three declined to participate, (“too tired” or “no desire to take part”). The final study population comprised five men and five women (see Table 1).

**Table 1:** Patient characteristics

Gender	5 men
	5 women
Age (years)	73 [51–79]
Indication for ICU admission	Acute respiratory failure/COPD: 3
	Cardiogenic pulmonary edema: 2
	Chest trauma: 1
	Acute respiratory failure/ALS: 2
	Pneumonia: 2^a^ (complicating lymphoma in 1 case)
Room air blood gases on admission or just before	
pH	7.34 [7.22–7.41]
PaO_2_ (mmHg)	63 [42–82]
PaCO_2_ (mmHg)	48 [36–74]^a^
Description of noninvasive ventilation	
Facial mask	All patients
Pressure support mode	All patients
FiO_2_ (%)	40 [30–70]
Pressure support level (cm H_2_O)	12 [8–24]
Positive end-expiratory pressure (cm H_2_O)	5 [4–9]
Number of NIV sessions before the interview	5 [4–7]
Description of the ICU stay^b^	
Duration (days)	6 [4–17]
NIV days	4 [2–11]

All numerical data provided as median [min − max]

*ICU* intensive care unit, *COPD* chronic obstructive pulmonary disease, *ALS* amyotrophic lateral sclerosis, *PaO*_*2*_ oxygen partial pressure in the arterial blood, *PaCO*_*2*_ carbon dioxide partial pressure in the arterial blood

^a^All patients were hypercapnic with PaCO_2_ > 45 mmHg, except 2 (PaCO_2_ 36 mmHg)

^b^All patients were discharged alive from the ICU; none were intubated during their stay; post-hospitalization home mechanical ventilation was instituted in three cases

All patients received NIV through a face mask held by an elastic harness that was chosen according to facial morphology and adjusted to minimize leaks. NIV sessions were discontinuous, their length depending on clinical monitoring and tolerance. NIV was delivered under the form of inspiratory pressure support, with positive expiratory pressure (see Table 1).

### Interviews

Semi-structured interviews were conducted at the bedside during the ICU stay, by a certified psychologist who had never met the patients before. The interviewer followed a guide designed for the study by two psychologists (of whom one had extensive experience of the ICU environment) and two intensivists (see Additional file 1). One interview per patient was performed. The patients were prompted to respond in four areas of interest coherent with those of the core “PARVENIR” study [5], namely, to elicit reflection in the following areas: (1) What did you feel before\during\after the NIV session? (2) What did you think before\during\after the NIV session? (3) What can you say about the caregivers during the NIV session? (4) What do you think about the presence of your relatives during the NIV session?

### Data analysis

All interviews were audiotaped and transcribed. We used evaluative assertion analysis [17] to measure the positive or negative aspects of attitudes toward NIV, and thematic analysis [18] to investigate the content of these attitudes phenomenologically. The text was divided into groups corresponding to the four themes of the interview. Each evaluative phrase was assessed for “connectors” between two components of an assertion, associative (e.g., “was”; positive score) or dissociative (e.g., “was not”; negative score). Connectors were scored from complete association/dissociation (+ 3 or − 3), to partial association/dissociation (+ 2 or − 2) to weak association/dissociation (+ 1, − 1). Phrases were also assessed for “evaluators” giving connotative meaning to attitudes, positive (e.g., “good”; positive score) or negative (e.g., “bad”; negative score). Evaluators were scored from extremely favorable/unfavorable (+ 3 or − 3), quite favorable/unfavorable (+ 2 or − 2) and slightly favorable/unfavorable (+ 1 or − 1).

The final evaluation score was transferred on a standard seven-point evaluation scale from + 3 (extremely positive attitude) to − 3 (extremely negative attitude), with 0 being considered as a neutral evaluation. The coding was performed by two certified psychologists familiar with these techniques, who had not conducted the interviews. In line with COREQ guidelines [15], the corresponding raw data are provided in Additional file 2.

To assess the reliability of the evaluative assertion analysis, we calculated the Spearman correlation coefficients between the scores attributed to “connectors” and “evaluators,” and we assessed the concordance of the direction of the evaluations (+ or −) between two experts. The scores obtained in the study population before, during and after NIV were compared using Friedman’s nonparametric analysis of variance, followed when relevant by a two-by-two Wilcoxon signed-rank test. A *p* value below 0.05 was considered significant. The Benjamini–Hochberg procedure was used to correct for multiple comparisons [19].

## Results

### Interviews

The interviews were conducted on the second day of NIV in five cases, and on the third day in five cases. Each lasted 27 min on average (range: 20–45). At the time of the interviews, all patients had a Richmond Agitation–Sedation Scale (RASS) score of 0 (“alert and calm; spontaneously pays attention to caregiver”) or 1 (“restless; anxious or apprehensive but movements not aggressive or vigorous”), acknowledging, however, that the RASS score is not designed to evaluate respiratory encephalopathy. SpO_2_ monitoring did not evidence significant desaturation during the interviews, with SpO_2_ values above 90% in all cases. No significant clinical event was noted.

### Reliability of evaluative assertion analysis

Inter-expert correlations were high for connectors and evaluators (0.940 and 0.978, respectively; *p* < 0.0001 in both cases). The experts diverged about the direction of connectors (positive or negative) in only 2.65% of cases, and about the direction of evaluators in 7.17% of cases.

### Cognitive attitudes

#### Before

**Fig. 1:**
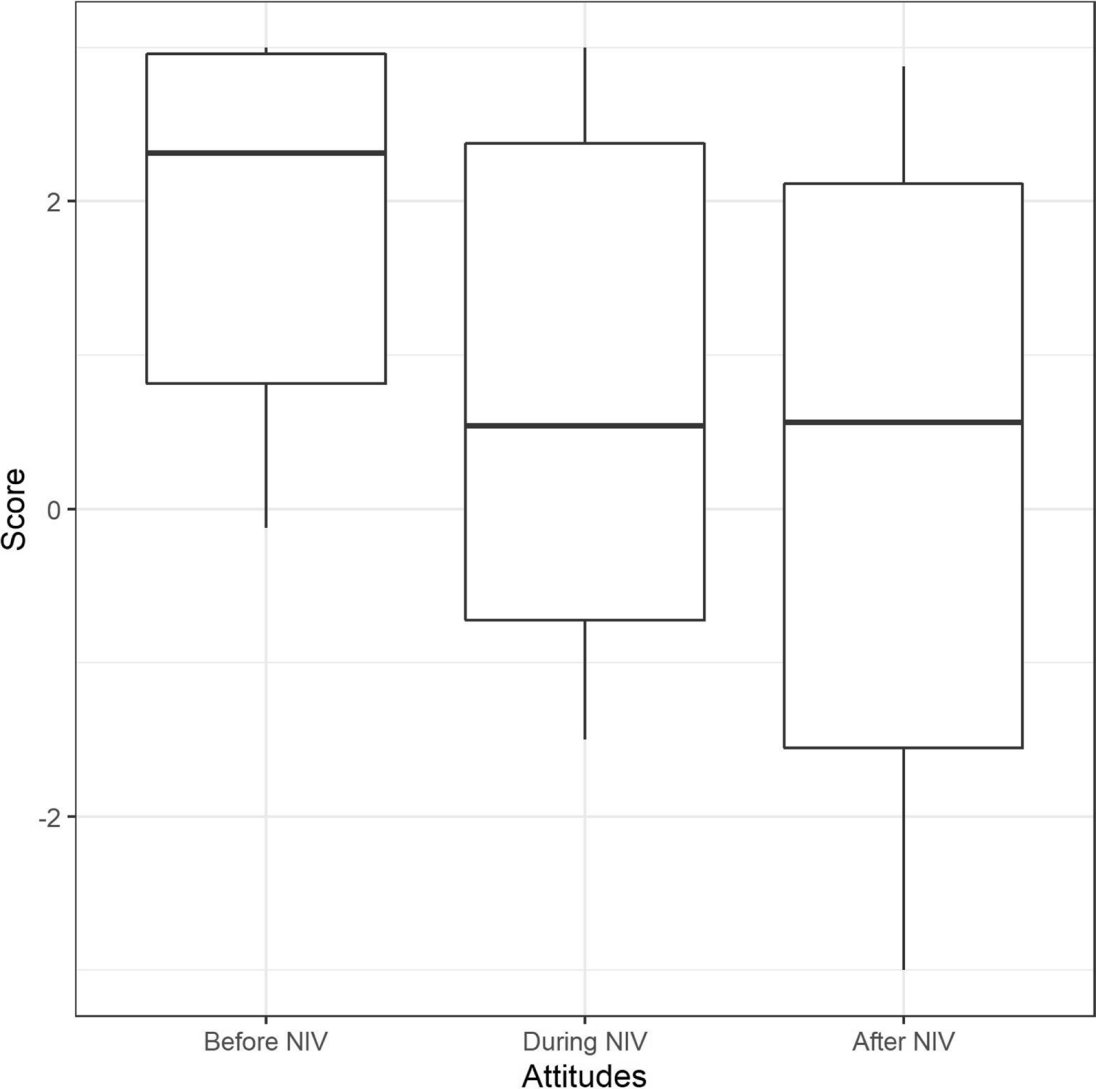
Cognitive attitudes toward noninvasive ventilation before, during and after the first noninvasive ventilation experience, according to the evaluative assertion analysis coding (see “Methods” for details). The maximum score of 3 denotes “extremely positive” attitude. The minimum score of − 3 denotes “extremely negative” attitude. The boxes represent the interquartile range (IQR) with indication of the median, the whiskers represent 1.5 * IQR. The observed differences did not reach statistical significance

Cognitive attitudes toward NIV prior to the first NIV session were positive in 9 of 10 patients (median = 2.31, IQR = 0.81–2.95) (Fig. 1). Eight patients reported they believed that this technique was supposed to improve their breathing. Three listed other expected benefits (improved breathing regularity; improved sleep; increased feeling of independence) and two possible barriers (novel experience with doubts about effectiveness; anticipation of a certain discomfort associated with the mask). The remaining two patients had no preconceptions and reported not having understood explanations about NIV or not having paid attention.

#### During

Cognitive attitudes toward NIV became less positive (median = 0.54, IQR = − 0.72–2.37) (Fig. 1). The eight patients who expressed positive attitudes “before” expressed doubts about the NIV efficacy “during”. Five mentioned mask-related pain and fatigue with a feeling of dependency. Six reported breathing discomfort attributed “to the ventilator” in two cases and “to themselves” in two cases, and fears of lacking air and dying. Only one patient reported no benefit at all (“*NIV induced dyspnea, and nothing else*”), while the others acknowledged some benefits. This led to ambivalent attitudes (for example: *It was awful,… It does make [breathing] better, but […]I’m not sure*) (full verbatim in Additional file 3). The two patients who did not report any particular cognitive attitudes toward NIV “before” reported a positive experience “during”.

#### After

Cognitive attitudes toward NIV “after” were less positive than “before” (median = 0.56, IQR = − 1.56–2.11) (Fig. 1). Two patients ruled out future use of NIV, considering disbenefits greater than benefits. One mentioned that sufferings endured during NIV were greater than those experienced during chemotherapy that he had past experience with. The others ascribed specific benefits to the treatment (increase in blood oxygen, improvement in sleep at night, recovery of a steady respiratory rhythm, and better independence) but reported NIV-related dyspnea, mask-related pain, lack of freedom, and communication.

#### Affective attitudes before

**Fig. 2:**
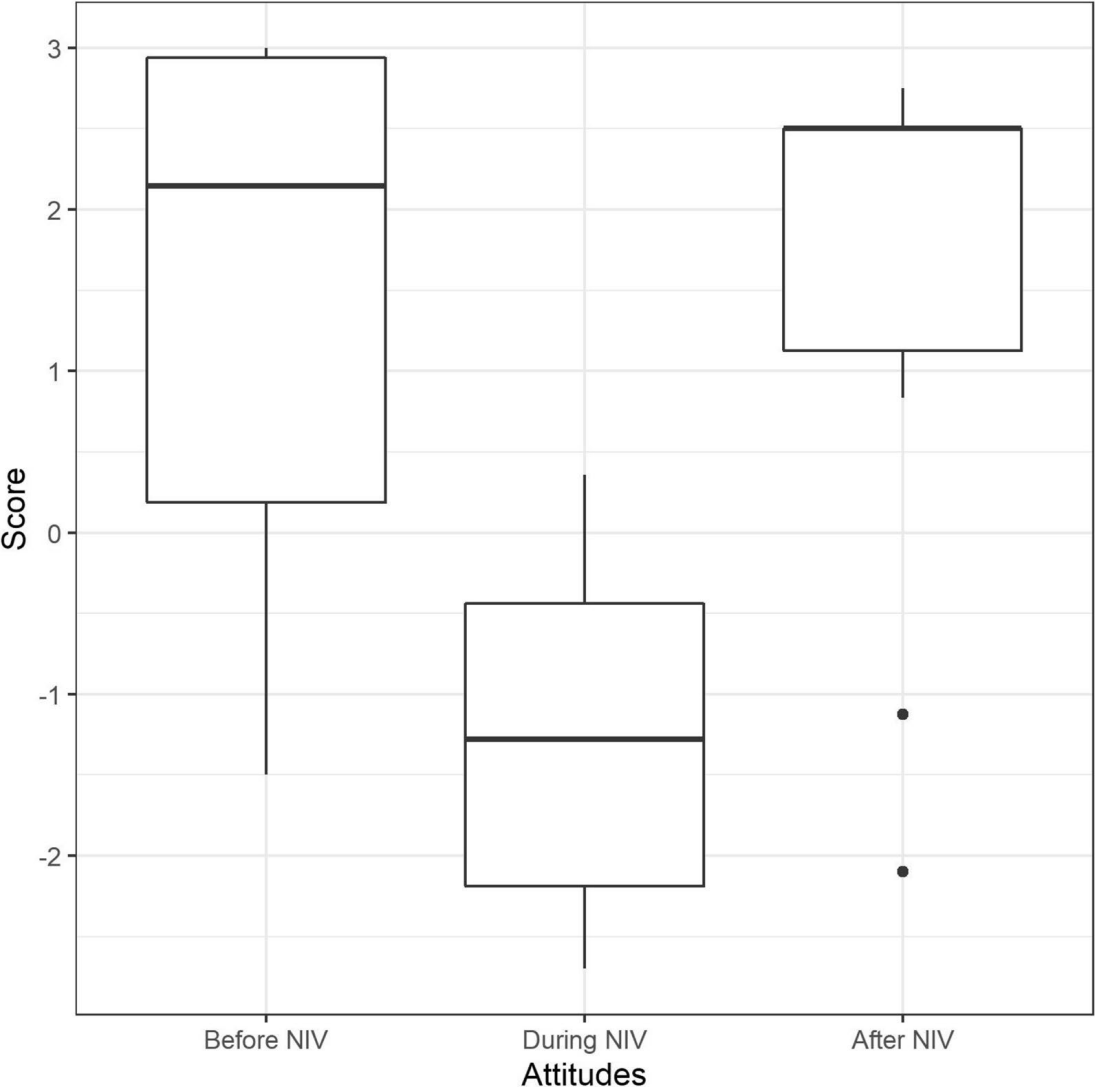
Affective attitudes toward noninvasive ventilation before, during, and after first noninvasive ventilation experience, according to the evaluative assertion analysis coding (see “Methods” for details). The maximum score of 3 denotes “extremely positive” attitude. The minimum score of − 3 denotes “extremely negative” attitude. The boxes represent the interquartile range (IQR) with indication of the median, while the whiskers represent 1.5 * IQR and the dots depict outliers. An overall statistically significant difference was detected between conditions (*χ*^2^ (2) = 10.400, *p* = 0.006) with a significant difference between “before” and “during” (*Z* = − 2.395, *p* = 0.017, with a large effect size (*r* = 0.536)), and between “during” and “after” NIV (*Z* = − 2.599, *p* = 0.009, also with a large effect size (*r* = 0.581)). There was no significant difference in between “before” and “after” (*Z* = − 0.561, *p* = 0.575, *r* = 0.125)

Affective attitudes toward NIV before the first NIV session were positive in eight patients and negative in two (median = 2.15, IQR = − 0.19–2.94) (Fig. 2). Eight patients reported that the prospect of receiving NIV was calming. They were not anxious and motivated. Two had already seen other people using NIV, which they considered reassuring. The two patients with negative affective attitudes had experienced anxiety upon seeing the machine (the prospect of having their face covered by the mask made them feel nervous). One patient was ambivalent: calm, not anxious, but disliking the machine.

#### During

Negative affective attitudes were noted in nine patients (median = − 1.28, IQR = − 2.19–0.44) (Fig. 2). Five reported anxiety related to dyspnea arising or worsening during NIV (*n* = 5), fear of dying (*n* = 1), pain caused by the mask, inability to move, feelings of loneliness, isolation and dependency (*n* = 3). They evoked lack of freedom, the impossibility of concentrating on anything other than their breathing, to communicate with other people (especially relatives). Two patients associated their NIV-related anxiety (taking the form of claustrophobia) with a prior healthcare-related traumatic experience (being tied to his bed during a childhood medical procedure, having a mask applied on the face during anesthesia induction) (“*It was at that time, the second session, that it came back to me… when I was ten I had an operation, and they put me to sleep with a mask, a bit like that one […] It gave me exactly the same feeling […] I had the feeling that I was trapped”)* (full verbatim in Additional file 3).

Only two patients did not describe negative affective attitudes toward NIV during the session. They reported breathing more easily despite temporary dyspnea and physical pain caused by the mask.

#### After

Affective attitudes “after” were positive in eight patients (improved breathing, *n* = 4; feeling of liberation, *n* = 2; feeling calmer, *n* = 3; more independent, *n* = 1) and negative in two (claustrophobic feelings) (median = 2.5, IQR = 1.12–2.5) (Fig. 2). It should be noted that the positive reports were related to withdrawal of the mask, and therefore may not actually depict positive reactions to NIV, but possibly the contrary.

### Attitudes toward caregivers during NIV

Five patients reported unambiguously positive caregivers attitudes, appreciating that caregivers came during sessions to ask them if they felt well, stayed with them for some time, instructed them about how to breathe during NIV (for example: *we came to an arrangement. I said to them, I’m expecting a visit so they would say OK, keep the mask on till then, then we’ll take it off and put it back on after your visitor has left)* (full verbatim in Additional file 3). Four patients reported ambiguous attitudes toward caregivers, emphasizing that in spite of giving important explanation and answering questions, some caregivers tended to impose the mask without any margin for “negotiation”, and tended to avoid entering the room during the NIV session. Finally, the tenth patient had an entirely negative attitude toward caregivers, who had imperiously insisted he put the mask on *(*for example: *The staff didn’t take it in. You’re having the mask, full stop! You’ve got to put the mask on, end of story!)* (full verbatim in Additional file 3).

### Attitudes toward relatives during NIV

**Fig. 3:**
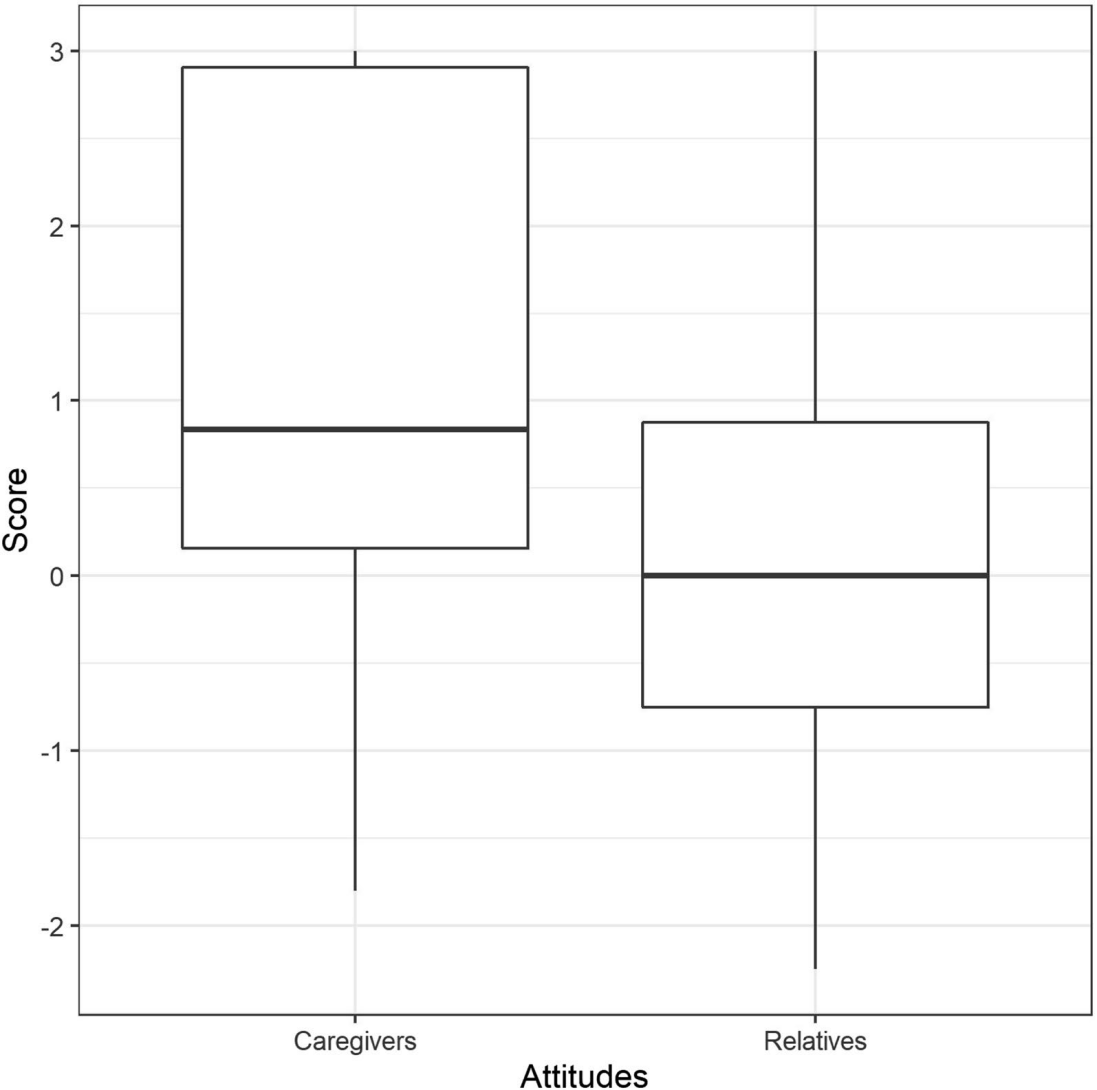
Affective attitudes toward caregivers and relatives during noninvasive ventilation, according to the evaluative assertion analysis coding (see “Methods” for details). The maximum score of 3 denotes “extremely positive” attitude. The minimum score of − 3 denotes “extremely negative” attitude. The boxes represent the interquartile range (IQR) with indication of the median, while the vertical bars 1.5 * IQR. There was a statistically significant difference between affective attitudes toward relatives and affective attitudes toward caregivers (*Z* = − 2.24, *p* = 0.025, with a large effect size (*r* = 0.501)

The attitudes of the patients toward the presence of their relatives in their room during the NIV sessions were significantly less positive than toward the caregivers (median = 0.12, IQR − 0.075–0.87) (Fig. 3). Only three patients reported completely positive attitudes. Six had mostly negative attitudes, explaining that they wanted to protect their family members from seeing them suffer (*n* = 3), or that they did not see the point of having their relatives present because they could not communicate with them anyway (*n* = 3). One patient was ambiguous *(Q: Does their [the family’s] presence reassure you? A: Hmm… it reassures me if they’re there. But it doesn’t reassure me when they see me fighting the mask. I have mixed feelings)* (full verbatim in Additional file 3).

## Discussion

This study confirms that NIV can be very trying for the patients even when clinically beneficial [5]. Indeed, cognitive and affective attitudes toward NIV markedly deteriorated during NIV. This is in line with the findings of the “PARVENIR” study, where a significant proportion of patients regretted having been selected to receive this treatment.

### NIV beliefs and attitudes

The patients generally expected NIV-derived benefits and did not describe anticipatory anxiety or fear, except in an abstract way (two patients). This raises the hypothesis that the information given by caregivers prior to NIV was mostly positive. This makes the failure of NIV to bring the anticipated benefits a source of frustration, worsening the traumatic nature of NIV through an “unpredictability” effect [8–10].

Prior beliefs (dyspnea relief, increased independence) were often contradicted by the actual experiences (persisting or worsening dyspnea, mask-induced pain, limited communication, or lack of independence), yet positive elements were also reported, leading to ambivalence. Ambivalence is known to make patient behavior unpredictable [20, 21]. This may lead to NIV being refused in spite of its putative benefits being acknowledged.

Our patients reported only negative emotions (anxiety, claustrophobic feelings, fear of death) likely stemming from vital threat combined with lack of control, a known source of post-traumatic manifestations: our observations are therefore perfectly consistent with reports of post-traumatic stress disorders after NIV-treated acute respiratory failure [22].Of particular importance, NIV awoke traumatic memories from the distant past in two of our patients, related both to breathing and to being tied and unable to move. We posit that this type of reaction can strongly contribute to the post-ICU psychological burden [22, 23]. Although positive remarks were made during the post-NIV period (improved breathing, freedom, independence and calmness), attitudes toward NIV were less positive after than before NIV. In fact, many of the positive remarks were associated with “taking the mask off”, suggesting that NIV cessation was considered, somewhat paradoxically, as a relief. All in all, our patients mentioned more barriers than benefits regarding NIV, which, according to the Health Belief Model, leads to predict NIV refusal [24]. Only two of our patients indicated that they would refuse NIV in the future. Mentioning barriers belonging to the affective register is in line with the findings of Baxter el al. [6] in patients with respiratory insufficiency due to amyotrophic lateral sclerosis.

### Caregivers and family members

Positive attitudes toward caregivers were associated with their perceived attention toward the needs of the patients while negative attitudes were associated with lack of dialogue. This emphasizes the importance of empathizing with the patients when implementing a treatment known as potentially stressful [25], something that is not always easy to achieve in the ICU high-burden environment [26, 27].

The attitudes of the patients toward the presence of their relatives during NIV sessions were generally negative, mostly because the patients feared that seeing them have breathing difficulties would distress their loved ones. This “intuitive” concern appears appropriate: being exposed to the respiratory suffering of a relative who dies during an ICU stay complicates grief and contributes to post-traumatic stress [28], in line with the recent demonstration that observing dyspnea in others induces a negative affect [29]. On the same plane, the avoidance behaviors of caregivers that some patients described could proceed from the suffering induced by the observation of NIV-related difficulties, possibly exacerbated by an impression of helplessness and hence of professional failure.

### Strengths and limitations

The major strength of our study lies in the novelty of the data provided: qualitative studies have addressed mechanical ventilation in the ICU [30–34], but none have focused on NIV in the context of acute respiratory failure. We acknowledge several limitations. Firstly, the population size is limited, and we did not formally evaluate data saturation [35]. Yet there were enough common elements in the discourses of the 10 patients to suggest that increasing their number would not have fundamentally changed the general pattern, and therefore to propose some conclusions [36]. Of note, the population under scrutiny was fragile and the study setting made both patient recruitment and the interviews highly demanding. In this regard, we acknowledge that the physiological conditions of the patients (e.g., intensity of gas exchange anomalies, intensity of dypnea) was bound to have an influence on their answers, and that this was not controlled in any sort of way. Secondly, our patient sample was homogeneous in some respects (hypercapnia in 8 cases, first contact with NIV in all cases), but heterogeneous in others (nature of the underlying disease, see Table 1). The majority of the patients enrolled suffered from chronic respiratory failure (COPD, ALS, left heart failure—Table 1) and only three suffered from de novo acute respiratory failure. Our data are therefore probably more pertinent to the former than to the latter situation. Thirdly, we only enrolled patients in whom NIV was considered a success after the first 24 h, namely not followed by tracheal intubation. This is evidently a possible source of bias, but it can be hypothesized that the experience of patients in whom NIV failed to avoid intubation should have been even worse. Fourthly, the interviews were conducted after NIV, so recollection biases regarding the “before” and the “after” periods are possible. It would have been methodologically superior to interview the patients before NIV and to repeat the interview after it, but this would have posed immense practical problems. Interviewing the patients during the NIV sessions is obviously impossible. However, conducting the interviews very soon after NIV initiation (rather than at the end of the ICU stay or after, as is often done in ICU qualitative studies), should have minimized recollection biases and preserved spontaneity. The delay between NIV start and the interviews could have influenced the nature of the patients’ responses, but this delay was balanced (5 patients interviewed on day 2, 5 on day 3). Finally, we did not follow the patients up and therefore cannot relate our observations to long-term physical or psychological outcomes.

### Propositions

Our observations help delineate propositions that may improve the NIV experience by preventing the development of negative attitudes that carry the risk of reducing patient concordance with the treatment [7, 37]. Firstly, caregivers should openly inform the patients, before the initiation of NIV, that it can provoke anxiety and has drawbacks that must be weighted against its benefits. In that, the appropriateness of the very term “noninvasive” appears questionable; “mask ventilation” probably better represents the reality of the treatment. Secondly, patients about to undergo NIV should be asked about past traumatic experiences, and informed that forgotten such experiences may be reactivated. Thirdly, the presence and availability of a caregiver able to empathize with the patient during NIV sessions seems an obvious necessity. This may call for specific training [38, 39], that may be based on simulation teaching or role play [40]. Fourthly, caregivers should enquire about dyspnea under NIV and to try to correct it [11, 41]. Fifthly, the presence of family members at the bedside should be discussed with the patient, as it can be counterproductive. Finally, it appears important that first NIV sessions be debriefed to avoid that further sessions “consolidate” a first traumatic experience. Some of these propositions can be implemented by ICU caregivers themselves, but others would be best implemented by a trained psychologist. Given the link between NIV poor tolerance and NIV failure and the corresponding negative outcomes, such measures could be of pronostic interest in addition to improving comfort.

## Conclusions

Cognitive and affective attitudes differed before, during and after NIV, the opinions of the patients tending to deteriorate with the experience. This study therefore corroborates the results of the quantitative “PARVENIR” study [5] and suggests that affective attitudes toward NIV strongly influence the nature of this experience. This is clinically relevant because affective attitudes strongly relate to health behaviors [42–44] and can produce deleterious dissociation between “clinical benefit” as evaluated by the physician and “life experience” as evaluated by the patient. We believe that our results provide a rationale for studies evaluating the impact of NIV-targeted psychological interventions in patients admitted in the ICU for acute respiratory failure and treated by NIV.

## Supplementary information

**Additional file 1.** Semi-structured interview guide designed to evaluate cognitive and affective attitudes towards non invasive ventilation in patients admitted ton an ICU for acute respiratory failure.

**Additional file 2.** Coding of the semi-structured interviews trancripts - integral raw data.

**Additional file 3.** French verbatims and their English translations of the excerpts from patients' interview quoted in the manuscript.

## Data Availability

All data generated or analyzed during this study are included in this published article and its additional files.

## Abbreviations

CAM-ICU: confusion assessment method for the intensive care unit (CAM-ICU)
ICU: intensive care unit
MV: mechanical ventilation
NIV: noninvasive ventilation
