# Supplementary material for: “I had the feeling that I was trapped”: a bedside qualitative study of cognitive and affective attitudes toward noninvasive ventilation in patients with acute respiratory failure
Source: Ann Intensive Care. 2019 Dec 2;9:134. doi: 10.1186/s13613-019-0608-6 (PMC6888797; doi:10.1186/s13613-019-0608-6)
Supplement: Supplementary file 1 — Additional file 1. Semi-structured interview guide designed to evaluate cognitive and affective attitudes towards non invasive ventilation in patients admitted ton an ICU for acute respiratory failure. [file 13613_2019_608_MOESM1_ESM.docx]

Cognitive and affective attitudes towards noninvasive ventilation: a bedside qualitative study in patients with acute respiratory failure.

Marina Iosifyan et al.

**Additional file 1**

*Semi-structured interview guide designed to evaluate cognitive and affective attitudes towards non invasive ventilation in patients admitted ton an ICU for acute respiratory failure.*

1. **Affective attitudes towards NIV**

- Can you tell, what did you feel about NIV?

**Affective attitudes before the first NIV session**

- What did you feel about NIV before the session, when you saw the machine but did not yet use it?
- What did you feel during the session?
- Can you name the emotions you felt during your first NIV session?

**Affective attitudes during the first NIV session**

- What did you feel about NIV during your first session?
- Did your feelings about NIV change when you started the ventilation?
- Why yes?
- Why not?

**Affective attitudes after the first NIV session**

- What did you feel about NIV when you finished your first session?
- Did your feelings about NIV change after your first session, as you took of the mask and finished the ventilation?

1. **Cognitive attitudes towards NIV**

- Can you tell me what words come to your mind when I say “non-invasive ventilation”, “ventilation”, “masque”?

**Cognitive attitudes before the first NIV session**

- What did you think about NIV before your first session?
- Did you think that NIV is effective, good for your health?
- Why yes?
- Why not?

**Cognitive attitudes during the first NIV sessions**

- What did you think about NIV during your first session?
- Did this experience change your opinion about NIV effectiveness for your health?
- Why yes?
- Why not?

**Cognitive attitudes during the first NIV sessions**

- What did you think about NIV after your first session?
- Did you change your opinion about NIV effectiveness for your health?
- Why yes?
- Why not?
- Do you think you should continue using NIV for better health?
- What are the benefits and barriers in using NIV?

1. **Attitudes towards caregivers during NIV**

- What can you tell me about the caregivers, the physicians, the nurses, during your NIV experience?
- Were the caregivers around you during your first NIV session? Would you prefer them to be nearby you more often?
- Did they give you some explanations and instructions concerning NIV?
- Do you think that the caregivers facilitated your first NIV experience?
- Why yes?
- Why not?

1. **Attitudes towards family members**

- Can you tell me about your family members during your first NIV experience?
- What do they think about NIV? Do they think it is good for your health?
- Do your family members think you should continue using NIV?
- Why yes?
- Why not?
- Were your family members around you during your first NIV experience? Would you prefer them to be nearby you during your NIV sessions?
