# Supplementary material for: “I had the feeling that I was trapped”: a bedside qualitative study of cognitive and affective attitudes toward noninvasive ventilation in patients with acute respiratory failure
Source: Ann Intensive Care. 2019 Dec 2;9:134. doi: 10.1186/s13613-019-0608-6 (PMC6888797; doi:10.1186/s13613-019-0608-6)
Supplement: Supplementary file 3 — Additional file 3. French verbatims and their English translations of the excerpts from patients' interview quoted in the manuscript. [file 13613_2019_608_MOESM3_ESM.docx]

Cognitive and affective attitudes towards noninvasive ventilation: a bedside qualitative study in patients with acute respiratory failure.

Marina Iosifyan et al.

**Additional file 3**

French verbatims and their English translations of the excerpts from patients' interview quoted in the manuscript.

| Excerpt N°  (in order of appearance) | French source | English translation |
| --- | --- | --- |
| 1  [from interview 2] | M. Et quand vous avez l’utilisé, vos émotions ?  P. J’ai trouvé ça perturbant. Ça fait mal, ça fait mal. Ça fait de bruit, ça... c’est fatiguant. On peut pas s’endormir avec cet appreil, même si ça fait mieux votre respiration. Par contre c’est vrai que ça avance, comment dire, vous vous demandez est-ce que ce n’est pas trop cher payé. Pour avoir les poumons, comment dire..  M. ça vaut pas la peine ?  P. Oui oui, voilà. Je ne suis pas sûr. | M. And when you had been on it [the ventilator], you felt... ?  P. It upset me. It was awful, awful. The noise, it's... it's tiring. You can't even get off to sleep when you're on the machine, even if it does make your breathing easier. It does make it better, but you do wonder—you know?—you ask yourself if it’s not too high a price to pay. And I'm not sure about that. To breathe better, you know...  M. It's not worth it?  P. That's it. I'm not sure. |
| 2  [from interview 1] | C’était la deuxième séance et à cette heure là, la deuxième séance, et puis me revenu à la tête le fait ..vous allez rire mais c’est comme ça ... le fait que quand j’avais dix ans j’étais opéré et on m’endormit avec le masque, un peu comme ça. A ça c’est revenu. Parce que après une fois j’étais opéré, une fois je suis sorti je pouvais plus voir le masque, rien, c’était fini. Ça m’a fait tellement souffrir, c’est dure, cette opération. D’ailleurs c’est long, deux heures...  M. A quelle moment vous avez eu cette mémore à propos de votre expérience traumatique ?  P. A peu près à la deuxième séance. Ça m’a donné exactement la même impression.  M. L’impression de quoi ?  P. L’impression...A l’époque on a vous attaché les pieds et les mains. Et bien j’ai eu cette impression de ne pas pouvoir faire ce que je voulais. J’ai eu l’impresson vraiment que je suis un prisionnier...du truc. Et après j’ai pas pu revoir, j’ai pas pu continuer.  M. Utiliser le masque ?  P. Avec le masque. J’ai du refaire trois ou quatre fois, je sais plus. Mais là après je ne pouvais plus voir. C’est fou... | It was the second session, at that time, the second session, that it came back to me... you'll think I'm joking, but that's how it was... when I was ten I had an operation, and they put me to sleep with a mask, a bit like that one [...] That's when it came back to me. Because after that operation, once I'd gone home I couldn't even look at a mask, never again. I really suffered, it was bad, that operation. And it's a long time, two hours [of NIV]...  M. At what point did you remember your previous traumatic experience?  P. Around the second session. It gave me exactly the same feeling.  M. What feeling was that?  P. The feeling... In those days they tied up your hands and feet. And so I had the feeling that I was trapped. I really felt imprisoned... by the thing. And after that I couldn't face it again, I couldn't go on.  M. With the mask?  P. With the mask. I was supposed to have it three or four times, I don't remember. But after that I just couldn't face it. It's crazy... |
|  | M. C’est les infirmières qui viennent vous mettre le masque  ?  P. Oui  M. Est-ce que parfois vous demandez d’enlever le masque  ?  P. Oui, ça peut m’arriver. Mais on a fait un bon accord. Je leur dit, par exemple, à telle heure je vais avoir une visite, et ils me disent alors dans ce cas la on garde le masque jusqu’a telle heure ou on l’enlève tout de suite et on remettra après. Donc on s’entend comme ça entre nous et ça se passe très très bien. | M. So it was the nurses who came to put on the mask?  P. Yes  M. Did you ask if you could take the mask off sometimes?  P. Yes, sometimes I did. But we came to an arrangement. I said to them, for example, I'm expecting a visit at a certain time, so they would say to me, OK, so in that case, keep the mask on till then, then we'll come in and take it off straightaway, and put it back on after your visitor has left. So we came to an understanding, and that went much, much better. |
|  | M. Et qu’est-ce que vous avez pensé à cette procédure pendant la ventilation ? Que ça vous aide, que c’est efficace ou pas ?  P. Dans un premier état non. Un jour ça se passe bien, y a pas de problème. Si j’ai des problèmes pour le mettre parce que je suis mal à x-raisons. Par exemple je tousse beaucoup, je crache beaucoup, il faut que je l’enlève et le remets et là ça fait la fuite et le pneumologue vous dit « vous avez fait une fuite » et je lui dit « mais je pouvais pas faire autrement parce qu’il faut que je crache, sinon je crache dans mon masque ». Voilà. Donc, là clairement il y a le discours avec les soignants qui ne comprennent rien.  M. Les soignants ont-ils expliqué à quoi ça sert le masque et comment l’utliser ?  P. Non.  M. Ils ont rien dit ? Juste donner le masque et parti ?  P. Oui.  M. Pendant la ventilation, étaient-ils à votre écoute ?  P. (crache) vous voyez qu’il y a un problème (crache) quand j’ai un masque (crache) c’est un gros problème.  M. Oui. Est-ce que le personnel soignant était à votre écoute pendant la ventilation ?  P. Les soignants ne s’ocuppent pas. Vous metttez le masque, point final ! Même eux, même ici.Vous devez mettre le masque , point ! Je trouve ça complétement stupide. Parce que si ils ont écouté d’une façon intélligente ce que j’ai à leur dire, parce que je leur dis au moment donné je sature avec ce masque, laissez moi cinq minutes simplément à réprandre un rythme normal et je reprends mon masque après. « Ah non ah non ah non ! » c’est quelque chose de .. je trouve ça complétement nul. | M. Did you think about this procedure when you were on the machine? Whether it was helping you, whether it was effective or not?  P. At first, no. On a given day it could go alright, no problems. If I have problems with putting it on, it's because I have plenty of reasons to have a problem. For instance, I cough a lot, I spit up a lot of phlegm, which means I need to take the mask off and put it back on again, and then that made it leak, and the specialist would say "you've made it leak" and I'd say, "but I can't help it because I've got to spit out the phlegm, or else I'll have to spit in the mask." There you have it. So, there, clearly, there's an example of staff who don't understand a thing.  M. Did the staff explain why you needed the mask, and how to use it?  P. No.  M. They didn't say anything? Just put the mask on and left?  P. Yes.  M. And when you had it on, did you feel they were attentive to you?  P. (spits) you see my problem (spits) when I've got the mask on (spits) it's a big problem.  M. Yes. Did you feel that the staff listened to your concerns when you were on the machine?  P. The staff didn't take it in. You're having the mask, full stop! Even the staff in here. You've got to put the mask on, end of story! I found that completely stupid. Because if they'd just listened sensibly to what I had to say, because at times I'd just had enough of that mask, just given me five minutes to sort myself out and I'll put the mask back on after. "Oh no. No, no you don't!" It's... something... I find it completely idiotic. |
| 5 | M. Est-ce que ça vous calme, leur présence ?  P. Bah, comment je peux vous expliquer...ça calme qu’ils soivent là. Mais ça me calme pas quand ils me voient bronchée. Quand j’ai paniqué à cause du masque ma mère était là et j’ai fait pleurer ma mère (commence à pleurer).  M. Vous ne voulez pas que vos proches vous voient quand vous êtes dans l’état...  P. Non.  M. Donc, si ça se passe bien vous aimez que vos proches sont avec vous, mais si ça va pas vous ne voulez pas ?  P. Oui. C’est contradictoire chez moi. Ils essayent de me protéger comme ils peuvent. Quand je suis arrivée le dimanche je ne savais même pas ce qui se passe.  M. Vos proches, qu’est-ce qu’ils pensent à propos de la ventilation ?  P. Bah, comment dire. Ils pensent que c’est très bien. Mais si c’est moi qui panique, c’est moi qui prend les décisions, donc...  M. Oui, mais ils pensent que c’est bien pour vous ?  P. Ils disent que ça améliore mon état. | M. Does their [the family's] presence reassure you?  P. Hmm, how can I explain it to you... it reassures me if they're there. But it doesn't reassure me when they see me fighting the mask. My mother was there when the mask made me panic, and that made her cry (sobs).  M. You don't want your family to see you in that state...  P. No.  M. So if things are going well, you like having your family around you, but if they're not, you don't?  P. Yes. I have mixed feelings. They try to protect me as best they can. When I arrived on Sunday, I didn't even know what was going to happen.  M. Your family, what do they think about the ventilator?  P. Hmm, not easy to say. They think that it's very good. But if it's me that's panicking, it's me who's deciding, so...  M. Yes, but they think that it's a good thing for you?  P. They say I seem better on it. |
